# Public values and guiding principles for implementing epitope compatibility in kidney transplantation allocation criteria: results from a Canadian online public deliberation

**DOI:** 10.1186/s12889-023-15790-w

**Published:** 2023-05-10

**Authors:** Caitlin Slomp, Louisa Edwards, Michael Burgess, Ruth Sapir-Pichhadze, Paul Keown, Stirling Bryan

**Affiliations:** 1 BC Children’s Hospital Research Institute, 938 W 28th Ave, BC V5Z 4H4 Vancouver, Canada; 2 Department of Psychiatry, University of British Columbia, Vancouver, Canada; 3 School of Population & Public Health, University of British Columbia, Vancouver, Canada; 4 Centre for Clinical Epidemiology and Evaluation, Vancouver Coastal Health Research Institute, Vancouver, Canada; 5 W. Maurice Young Centre for Applied Ethics, University of British Columbia, Vancouver, Canada; 6 Division of Nephrology, Department of Medicine, McGill University, Montreal, Canada; 7 Centre for Outcomes Research & Evaluation, Research Institute of the McGill University Health Centre, Montreal, Canada; 8 Department of Medicine, University of British Columbia, Vancouver, Canada; 9 Immune Centre of BC, Vancouver Coastal Health, Vancouver, Canada

**Keywords:** Public engagement, Public values, Public policy, Bioethics, Precision medicine, Epitopes, HLA, End-stage kidney disease, Kidney transplantation

## Abstract

**Background:**

Epitope compatibility in deceased donor kidney allocation is an emerging area of precision medicine (PM), seeking to improve compatibility between donor kidneys to transplant candidates in the hope of avoiding kidney rejection. Though the potential benefits of using epitope compatibility are promising, the implied modification of deceased organ allocation criteria requires consideration of significant clinical and ethical trade-offs. As a matter of public policy, these trade-offs should consider public values and preferences. We invited members of the Canadian public to participate in a deliberation about epitope compatibility in deceased donor kidney transplantation; to identify what is important to them and to provide recommendations to policymakers.

**Methods:**

An online public deliberation was conducted with members of the Canadian public, in which participants were asked to construct recommendations for policymakers regarding the introduction of epitope compatibility to kidney allocation criteria. In the present paper, a qualitative analysis was conducted to identify the values reflected in participants’ recommendations. All virtual sessions were recorded, transcribed, and analyzed using NVivo 12 software.

**Results:**

Thirty-two participants constructed nine recommendations regarding the adoption of epitope compatibility into deceased donor kidney allocation. Five values were identified that drove participants’ recommendations: Health Maximization, Protection/Mitigation of Negative Impacts, Fairness, Science/Evidence-based Healthcare, and Responsibility to Maintain Trust. Conflicts between these values were discussed in terms of operational principles that were required for epitope compatibility to be implemented in an acceptable manner: the needs for Flexibility, Accountability, Transparent Communication and a Transition Plan. All nine recommendations were informed by these four principles. Participant deliberations were often dominated by the conflict between Health Maximization and Fairness or Protection/Mitigation of Negative Impacts, which was discussed as the need for Flexibility. Two additional values (Efficient Use of Resources and Logic/Rationality) were also discussed and were reasons for some participants voting against some recommendations.

**Conclusions:**

Public recommendations indicate support for using epitope compatibility in deceased donor kidney allocation. A flexible approach to organ allocation decision-making may allow for the balancing of Health Maximization against maintaining Fairness and Mitigating Negative Impacts. Flexibility is particularly important in the context of epitope compatibility and other PM initiatives where evidence is still emerging.

**Supplementary Information:**

The online version contains supplementary material available at 10.1186/s12889-023-15790-w.

## Background

Precision medicine (PM) is becoming increasingly relevant and potentially applicable to a growing number of medical specialties [1], with the goal of individualizing treatment in order to maximize therapeutic benefit and avoid unnecessary or ineffective treatment. PM is often based on rapidly expanding and evolving knowledge and, given the balance of risks, costs and potential benefits, requires careful appraisal of the criteria and policies used in clinical decision-making [2]. These decisions can prioritize prognosis (seeking to treat those with the greatest potential to benefit; utilitarian-based) or diagnosis (allocating resources to patients with the greatest need; justice-based) [2]. PM initiatives must carefully consider the trade-offs between these approaches and the resulting ethical implications. The application of PM technologies is funded largely through public funding, with implications for who benefits and who does not; members of the public are therefore key stakeholders, whose values and preferences will affect the uptake and impact of PM initiatives [1, 3, 4]. Public participation, in a transparent and accountable manner, will increase the trustworthiness of decisions and the health systems they support; it is, therefore, imperative to understand and include public values into the delivery of PM [4, 5].

An emerging area of PM is the use of epitope (or molecular) compatibility in deceased donor kidney allocation. Epitope compatibility compares targeted segments of Human Leukocyte Antigens (HLAs) between donors and potential recipients. Epitopes represent immune markers that are used to distinguish the body’s own cells from foreign cells [6]. Comparing epitopes enables an estimation of the degree of match, or mismatch, between a donor and recipient and, potentially, more accurate assessment of a transplant candidate’s immune risk post-transplantation. Projected benefits of using epitope compatibility for kidney allocation include reduced risk of rejection, improved graft function and longevity, and decreasing the need for re-transplantation [7]. Consideration of epitope compatibility is promising, but it is not yet routinely used in kidney allocation programs [7]. Prior to implementing this change to the allocation criteria, societal and ethical questions must be addressed; specifically, the trade-off between maximizing the utility of a transplant, and ensuring equitable access to transplantation. Maximizing the utility of each transplant using epitope compatibility could benefit both the kidney transplant recipient and the transplant community as a whole through improved graft survival and more efficient use of scarce resources. However, the use of epitope compatibility could result in extended waiting times for some candidates [6] and/or inequitable access to transplants due to difficulty matching certain groups of patients [7].

The trade-off between utility (selecting a recipient based on the probability of successful transplant) and equity (ensuring fair access to organs) has been discussed often in the deceased organ allocation literature [6, 8–10]. Organ allocation policies have typically favored equity [9, 10], while a shift to epitope compatibility would imply an increasing interest in PM tools and utilitarianism [9].

While attempting to achieve balance between these ethical principles regarding the use of limited donor organs (managed, in most jurisdictions, as a publicly-held resource), it is important to consult and include public preferences and values in organ allocation programs [6, 11–13]. Public preferences for deceased organ allocation have been reported from Germany [11], Australia [12], the UK [13–15], USA [14, 15], Hong Kong [16], Iran [17], and India [18]. In general, participants endorsed the prioritization of transplant candidates with the most potential for successful outcomes [11, 12, 14–17], as well as those with medical urgency [11–13, 15]. However, there is a lack of evidence on public preferences regarding the trade-offs between different allocation decisions [11, 12], and no reports on Canadian public preferences for kidney allocation. Furthermore, public preferences for the use of epitope compatibility to guide deceased donor kidney allocation, and the values underlying these preferences, have yet to be explored.

We, therefore, invited members of the Canadian public to participate in an online public deliberation about epitope compatibility in deceased donor kidney allocation; to identify what is important to them and provide recommendations to policymakers. The results of this deliberation, including the final list of nine recommendations and participant vote counts for each recommendation, have been published elsewhere (Edwards et al.*,* under review). Participants were supportive, but struggled to agree on the specifics of how exactly epitope compatibility should be incorporated into kidney allocation criteria; they felt that specific details should be determined by clinical or policy experts. The purpose of the present qualitative analysis was therefore to extend our earlier work and to provide further guidance for the development of kidney allocation programs using epitope compatibility, in a manner that upholds public values. Specifically, we explored 1) the values reflected in public discussions about the implementation of epitope compatibility for kidney allocation, and 2) the relationship between participant values and the recommendations formed through the public deliberation process.

## Methods

### Deliberative public engagement

A public deliberation is a process through which socially diverse members of the public are informed about a given topic, engage with one another to discuss various issues related to that topic, and then collectively form recommendations for future policy formation [19]. Public deliberations are particularly useful in settings with regulatory or ethical uncertainty; they invite participants to consider and engage with alternative values, perspectives or positions in order to expand one’s knowledge and contribute to the collective decision-making process (a form of deliberative democracy) [20].

Details about the deliberative public engagement are described elsewhere (Edwards et al., under review [21]); an overview is provided here. A virtual deliberation (modified from a 4-day in-person deliberation [22, 23] and developed with input from methodologists, bioethicists, researchers, clinicians, patient partners and policymakers), was held by Zoom from November–December 2021. Participants were recruited via postal invitation to randomly selected households in all provinces and territories in Canada. Interested individuals completed an online recruitment survey, and eligible participants were selected to ensure a range of key socio-demographic variables of interest (gender, age group, ethnicity, religion, and urban/rural location) and to represent each of the five Canadian regions (Western, Prairies, Central, Maritimes and Northern Territories). We excluded anyone with kidney disease or who had someone close to them with kidney disease, those who worked or volunteered for a kidney disease organization, health professionals, policymakers, and lobbyists. Prior to the deliberation, participants were provided with a detailed information booklet to ensure a foundation of sufficient and balanced information to inform their discussion. The booklet included background information on kidney disease and transplantation, as well as potential advantages and concerns with epitope compatibility.

Five online, 2-h sessions were conducted. All sessions were facilitated by a trained and highly experienced facilitator, who identified areas of consensus and disagreement throughout the deliberation. In accordance with public deliberation methods [20, 22], participants were asked to justify and respectfully challenge stated positions and to explore points of disagreement further. Notes were recorded by a designated member of the study team. Participants were asked to deliberate and form recommendations regarding the use of epitope compatibility-guided allocation by considering two main questions: 1) How can allocation cognizant of epitope-compatibility be implemented in a way that is fair for transplant candidates? and 2) What are important considerations in the way kidney allocation policies and decisions are made? The five sessions proceeded as follows (see supplementary material for further details):

- Session 1 (all participants): Information session with five experts (transplant nephrologist, bioethicist, Indigenous elder and knowledge keeper, and two patients with kidney disease). The speakers represented a range of perspectives and spoke about the trade-offs and ethical considerations in changing kidney allocation criteria.
- Session 2 (four small groups, 7–8 participants each): Small-group discussion to identify participants’ different perspectives, beliefs and values through discussing their hopes and concerns of epitope compatibility-guided allocation.
- Sessions 3–4 (all participants): Discussion of the two deliberation questions and construction of recommendations.
- Session 5 (all participants): Final deliberation and review of recommendations, and discussion with four policy panelists from Canadian kidney organizations (BC Transplant, Trillium Gift of Life, Transplant Quebec, Canadian Blood Services).

This study was approved by the University of British Columbia Behavioural Research Ethics Board (#H21-01254) and McGill University Health Centre Research Ethics Board (#2022–8196).

### Qualitative analysis

In a public deliberation, participants’ knowledge and statements evolve over time, with the express purpose of collectively forming and voting on recommendations [23]. Because of the unique nature of the resulting data, straightforward thematic or content analysis is not applicable nor appropriate [19]. Our analysis focused specifically on the values reflected in participants’ discussions of and recommendations for introducing epitope compatibility into kidney transplant allocation decisions, with increasing significance accorded to statements made later in the deliberation process [19].

Each online session was recorded and transcribed, then imported into NVivo 12 [24] for analysis. Line-by-line coding was conducted for all transcripts containing participant discussion (sessions 2a-d, 3, 4, and the first hour of session 5), using both deductive and inductive approaches. To begin, and to focus our analysis on values related to healthcare decisions, codes for ethical principles utilized in public health [25] were created and applied to data deductively. As deductive coding did not fit our data entirely and inherently limits which data are included in further analyses, inductive (open) coding was also used to capture additional ideas and values that were evident in the transcripts. Definitions of value codes – including those which were initially identified by the authors and deductively applied—were inductively developed from the data in order to maintain the notion of each value as discussed by participants, rather than using definitions from the literature. Two coders (CS [MSc, female, with 6 years of professional quantitative and qualitative research experience] and LE [PhD, female, with 12 years of professional quantitative and qualitative research experience]) independently coded session 3, which was selected as a source of rich data. Coding discrepancies were resolved through discussion until consensus was achieved, and a codebook was developed and applied by CS to remaining transcripts (see supplementary material). The codebook and value definitions were iteratively modified as needed through group consensus between CS, LE and SB. LE attended all sessions and interacted regularly with deliberation participants leading up to and throughout the online event (recruiting, communicating with participants via email with session reminders and materials, scribing for small group sessions [sessions 2a-d]). SB attended the first and last sessions (1 and 5), provided participants with the deliberation overview, and facilitated the final discussion between participants and policymakers. CS had no interaction with participants, but was a silent observer at four sessions (2a, 3–5).

Axial and hierarchical coding were used to identify main concepts and the relationships between them. CS, LE and SB met weekly for two months to discuss main concepts and patterns and to resolve potential issues, until participants’ values, the conflicts between them, and their influence on the discussion and resulting recommendations were identified. Memos were used to capture decision-making, and additional documents (the complete list of recommendations and reasons for participants’ votes) were referenced throughout the analytical process. Pseudonyms are used to protect participants’ identities.

## Results

### Overview

Thirty-two participants took part in the deliberation; demographic information is shown in Table 1. The majority of participants were White, born in Canada and had post-secondary education. There were slightly more female than male participants. Nine recommendations were constructed and voted upon by participants (see Table 2, and Edwards et al. (under review) for further details of the recommendations and outputs of the deliberation).

**Table 1:** Socio-demographic characteristics of deliberation participants (*N* = 32)

Characteristic	*N* (%)
Gender	
Female	18 (56)
Male	14 (44)
Ethnic background^a^	
White/European	23 (72)
Arab	3 (9)
Indigenous	2 (6)
East Asian	2 (6)
South Asian	1 (3)
Latin, South or Central American	1 (3)
Region	
West Coast	5 (16)
Prairie Provinces	10 (31)
Northern Territories	1 (3)
Central Canada	11 (34)
Atlantic Provinces	5 (16)
Country of birth	
Canada	30 (94)
Other	2 (6)
Age group (years)	
18–24	4 (13)
25–34	3 (9)
35–49	9 (28)
50–64	10 (31)
65 +	6 (19)
Highest level of education attained	
High school diploma/certificate	3 (9)
College/apprenticeship (non-university)	4 (13)
Some university	8 (25)
University degree or diploma (BA/BSc level)	11 (34)
Professional or graduate degree	6 (19)
Main activity	
Working at a paid job/business	18 (56)
Retired	8 (25)
Looking for paid work	2 (6)
Going to school	2 (6)
Household work	1 (3)
Long-term illness	1 (3)
Chronic condition (personal or dependent)	
Yes	8 (25)
No	24 (75)
Income ($ CAD)	
Less than $20 K	3 (9)
$35 K-$49,999	2 (6)
$50 K-$79,999	6 (19)
$80 K-$99,999	9 (28)
$100 K +	12 (38)
Religion	
Christian (United, Baptist, Anglican, Catholic)^b^	17 (53)
No religion	12 (38)
Hindu	1 (3)
Aboriginal spirituality	1 (3)
Muslim	1 (3)

*NB* Percentages may not always sum to 100% due to rounding

^a^Categories are based on the Canadian Census categories for ethnic origin

^b^Denominations of Christianity were asked separately, but have been grouped here for ease of presentation. Religious categories were based on the Canadian Census categories

**Table 2:** Recommendations and distribution of participant votes

Recommendation	Y	N	A
1. Epitope compatibility should be added as an additional criterion (added to the matrix) for transplant candidate selection	30	0	1
2. Safeguards/flexibility need to be part of epitope compatibility to promote fairness	28	0	3
3. When epitope compatibility is being considered, we should also allow people with seriously declining health to receive less- or non-epitope matched kidneys.^a^	23	3	1
4. Quality of life should be considered as a priority	11	12	7
5. Deteriorating health should be considered as a priority	20	5	5
6. Epitope matching should be given high, but not absolute priority in the allocation of kidneys	29	0	1
7. There needs to be an ongoing comprehensive education program for the public, beginning with the transition to epitope matching	27	1	2
8. There needs to be a transition period and plan before starting the epitope matching system.^b^	25	0	2
9. Assessing epitope compatibility outcomes at least every 5 years and communicate results widely to patients, healthcare professionals, and public, whether successful or not	29	0	0

*Y* Yes, *N* No, *A* Abstain

^a^Session went overtime and 4 participants were unable to stay longer and vote

^b^Session went overtime and 3 participants were unable to stay longer and vote

Our analysis identified two domains of results: first, the values that were important to participants and were reflected in their deliberations. Second, operational principles that were expressed as requirements for the implementation of epitope compatibility in an acceptable manner. The operational principles served to uphold participants’ values and directly informed the construction of the nine recommendations.

### Participants’ values

Early discussions (sessions 2a-d) generally centered around participants’ perceptions of the potential benefits and negative impacts of introducing epitope compatibility to kidney allocation decisions. Participants hoped for benefits, such as improved health for transplant recipients, and expressed concern about the possibility of physical or psychological distress for patients waiting for transplants. As discussions progressed throughout sessions 3–5, participants began considering the trade-offs between various courses of action and which outcomes should be prioritized. These discussions, and the construction of recommendations, compelled participants to articulate which values they felt were important and worthy of consideration. While numerous values could be identified at various points the deliberation (see supplementary material), five values drove much of the discussion: Health Maximization, Protection/Mitigation of Negative Impacts, Fairness, Science/Evidence-based Healthcare, and Responsibility to Maintain Trust (see Table 3). Two additional values were identified that influenced participants’ voting on recommendations: Efficient Use of Resources, and Logic/Rationality. Although these values were discussed in the context of kidney transplantation, they were general in scope and were potentially applicable to other areas of public healthcare.

**Table 3:** Definitions and illustrative quotes of the values identified from participants’ discussions about using epitope matching for kidney transplant allocation

Value	Definition	Illustrative Quote
Health Maximization	Pursuit of the most health benefit for the most people (utilitarianism); improving public/population health	“We have to look at it on the bigger picture in terms of the long-term benefit to the entire transplant community [versus individual concerns and impact].” – David, session 3 “Because of the limited number of kidneys, there's always going to have to be a point at which somebody decides, you get it and you don't. And to me, it seems the fairest way to decide is, who is going to be best matched with that kidney? And then we're not going to have to come back in five years, and put another kidney in that person.” – Peter, session 2d
Protection/Mitigation of Negative Impacts	The imperative to put in place strategies to protect the vulnerable, or against possible negative effects	“You know you’re going to reach this tipping point. Let’s put it rather colourfully by saying a person might be ready to drop dead. At some point I kind of agree that there maybe needs to be a trigger to say hey, wait a minute, we need to bump this person to the top because they’re really in a bad state OK.” – Dan, session 3
Fairness	The principle of standardized treatment such that no person is unduly favoured or disadvantaged	“I can be the fifth in line at the grocery store with a carton of milk, but I have three or four people in front of me who have a hundred items. I still have to stand in line. So I can’t just jump to the front of the line because all I have is a carton of milk.” – Sara, session 3
Science/Evidence-based Healthcare	The goal or obligation of using of health interventions that have been shown to result in benefit for patients	“[I hope] that there’d be a large body of research that shows – that actually supports the claim or proved outcomes. Because right now, if you can’t say, you know, we tried this for 20 years, with thousands of people, and this is what it comes out to, short of that you have to talk people into it, because you’re not using actual experience to support that.” – Colleen, session 2C “I think it’s important to recognize like if epitope compatibility is the science, right, as opposed to blood types, then it underlies this anyway. And the question is not whether or not it exists, but whether or not you’re measuring it. And I find, so if that is the science I find it hard to say we’re just not going to measure it, we’re not going to use it.” – Brandon, session 3
Responsibility to Maintain Trust	Responsibility of decision-makers/healthcare systems to act out of respect for the public, to ensure health programs meet patient needs and expectations	“There needs to be buy in. I mean, you know, people’s lives are on the line. And so any transition, you would need a lot of public awareness and understanding of benefits and stuff; because there’s so much potential to do damage in immediate moment.” Colleen, session 2c
Efficient Use of Resources^a^	Moral duty to use scarce health resources in the best possible way	“So, what it comes down to is—reducing the need for a second or third transplant and extending the life of an existing transplant, works very much to control the use of disposable resources.” – John, session 2d
Logic/Rationality^a^	The use of a scientific, logical, objective or otherwise rational approach to decision-making	“I guess when you’re making decisions and if you introduce subjectivity into it then it’s kind of a slippery slope maybe versus […] clinical reasoning.” – Michelle, session 4

^a^These values did not contribute to operational principles; however, they were discussed multiple times by participants and were cited as reasons for voting against some recommendations by several participants

The value of Fairness was a recurring point of discussion, and was referenced by participants in multiple contexts, such as fairness for people who had already been on the transplant waitlist for some time; fairness for people with less common epitopes; fairness for people living in rural areas or with less access to healthcare centres:

Sandy (session 2b): My big concern would be is someone has been waiting for such a long time and they cannot seem to get that match […].

Gabriel: Yeah.

Alicia: Yeah.

Jacques: I think yes, it’s a concern… I mean I know it’s not fair for someone who has an epitope that doesn’t match, but life is not fair… I wouldn’t want it to be unfair in the sense that you know, give kidneys to women and not give them to men, or vice versa. Or something like that. But if it’s because your epitope causes you your problem, well it’s not fair for those who have kidney problems, no? Life is not fair.

What was meant by “fairness” was not often explicitly defined by participants, and seemed to be subjectively perceived and applied. For example, to some participants it was not fair for a person to have to wait longer than others; for other participants, it was not fair for a person to be prioritized based on wait time over someone with more medical urgency. Participants acknowledged that fairness did not necessarily mean equal treatment, and that in certain circumstances, it may be considered fair for someone to receive special consideration.

Gabriel (session 3): If they’re sick and they’re going to die soon, well they need, it’s only equitable that their life be maintained. So, you look and you do an exception for them, but it’s not the general rule. The general rule is you use epitope [compatibility] or you use this or that. But in this specific case you make the exception.

Participants differed, however, in terms of the circumstances under which they believed special consideration was warranted. For example, some participants thought an exception was justified for a person waiting 10 or 15 years for a transplant, while others disagreed as long as the patient could still tolerate dialysis. Some participants also suggested special consideration for patients who lived remotely and needed to travel for dialysis, or for patients in other specific circumstances that would impact their quality of life.

The perspectives of several participants evolved over the course of the deliberation, such that something they initially expressed was unfair (e.g., a longer than average wait time) was later considered acceptable to ensure fairness in another sense (e.g., access to a kidney before one’s health has declined to the point of medical urgency). Thus, the term “fairness” contained significant complexity, despite being frequently used. The commonality between the various usages of the word fairness was the principle that no individual should be favoured or disadvantaged without good reason (see Table 3).

### Operational principles

**Fig. 1:**
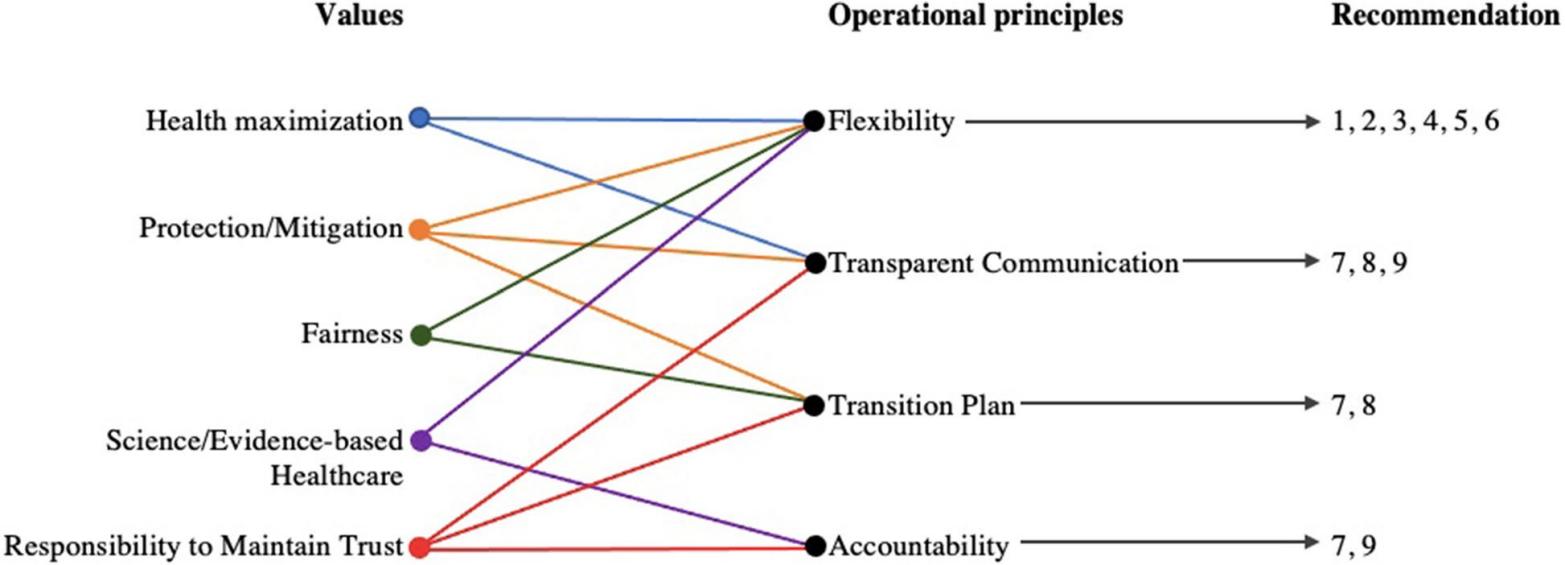
Influence of participants’ values on operational principles that drove recommendations for introducing epitope compatibility to kidney transplant allocation. Values were often discussed indirectly as operational principles, which served to uphold several underlying values. Operational principles then directly drove participants' discussions and ultimate recommendations for introducing epitope compatibility. See Tables 2 and 3 for definitions

As the deliberation progressed, more complex discussions arose in which participants’ values were in conflict with one another. These tensions were not typically discussed explicitly as ‘value conflicts’; rather, participants discussed various operational principles that should be incorporated into plans for introducing epitope compatibility in an acceptable manner. These principles – the needs for Flexibility, a Transition Plan, Transparent Communication, and Accountability – moderated how participants’ values could be upheld or balanced against one another (Table 4). Although driven by values that were general in nature, these four operational principles were discussed in terms specific to the epitope compatibility/kidney transplantation context. In this way, these needs operationalized participants’ values in ways that could affect kidney transplant policy, and fed directly into participants’ final recommendations for epitope compatibility (Fig. 1). Each operational principle was informed by two or more values, either as a way to maintain multiple complementary values or to balance conflicting values against one another. For example, the need for a Transition Plan could uphold Fairness, Protection/Mitigation of Negative Impacts, and Trust in the healthcare system. The need for Flexibility could uphold Fairness and Protection/Mitigation of Negative Impacts, while also balancing Health Maximization and the need for Evidence-based care (discussed below in detail).

**Table 4:** Operational principles driving participants’ discussions about how to implement epitope matching for kidney transplant allocation

Operational principle	Definition	Underlying Values	Illustrative Quote
Need for Flexibility	Desire for safeguards or exceptions built into the epitope-matching system to help mitigate potential negative effects; to introduce epitope testing in a cautious or “balanced” way when potential benefits are not guaranteed	Protection/mitigation of negative impacts Fairness Science- or evidence-based healthcare Health maximization	“There’s multiple factors that people want to play into when they get a kidney, and one of them would be age and one of them might be years on dialysis [or dialysis health] and one of them might be how good the epitope matching is…. And so I think using a system where it’s an algorithm where it combines all these factors, that would allow the flexibility and take into account all the factors we think are important.”– Adam, session 3
Need for a Transition Plan	Desire for a slow and well-planned shift from the current kidney matching system to the epitope compatibility-informed system, to help mitigate negative impacts and help patients on the waitlist adjust to the change	Protection/mitigation of negative impacts Fairness Responsibility to maintain trust	“I think it would be pretty shocking for people to be anticipating a new kidney in the next 24 months or 28 months to all of a sudden be said, well it could easily be 10 years from one day to the next. That they be advised that in six months or in a year and a half, whatever is necessary, for people to get used to the idea that the system has changed. You need a transition period I believe.” – Henry, session 4
Need for Transparent Communication	Desire for clear and honest communication with healthcare users about the rationale, planning process and potential impact of the switch to an epitope-matching system to mitigate negative effects and maintain trust in the transplant system	Protection/mitigation of harm Health maximization (through increased kidney donation) Responsibility to maintain trust	“I thought about transparent communication. So acknowledging that there’ll be some people that it might be different for them with the change… Transparency but make it human too because these are people’s lives, right.” – Anne, session 4
Need for Accountability	Need to ensure that the new epitope-based matching system performs as expected/promised; and/or that adjustments or changes be made in the event that epitope matching does not result in net benefit	Responsibility to maintain trust Science- or evidence-based healthcare	“So there's a lot of data, there's a lot of statistics on how it's working, or not working. I would hope that there would be some sort of mechanism in the adoption if we proceed that way to monitor the epitope compatibility to ensure its success, and it is actually improving the allocation program. And then could be modified or adjusted if that's not the case.” – Anne, session 2b

For several sessions, the majority of participants’ conversation was dominated by the conflict between Health Maximization versus Fairness and Protection/Mitigation of Negative Impacts. This was discussed at length as the need for Flexibility: built-in criteria or exceptions that would ensure that some degree of Protection/Mitigation and Fairness was ensured while epitope-based matching was implemented. The following excerpt from session 4 demonstrates the tension between ensuring Fairness for people waiting for a transplant for long periods of time, versus trying to protect transplant candidates with declining health from serious negative impacts such as loss of life:

Sara: I agree with what's being said about having a score [to calculate candidates’ priority for a transplant] and time on the waiting list isn't necessarily that important, but at the same time, I do think like if you have somebody who can handle dialysis for 10 to 15 years, they actually get punished for being in better health.

Facilitator: Interesting.

Sara: You know, so they're constantly getting bumped because somebody else is sicker than they are or, you know.

Anne: That's a good point.

Denise: I agree with [Sara]. I totally agree with what she's saying, and I don't think you're ever going to find something that's fair.

Evelyn: Yeah. But like, that's when you go to the emergency room, right? Like everybody is sick and needs to see a doctor. But the ones that are more critical are going to be seen to first, right? … My son is not going to die if he doesn't get seen by the doctor because he has a broken arm versus somebody who's coming in with cardiac arrest. And so, yeah, like, it isn't fair. I agree. It's not fair for somebody who's been waiting on the list. But I think we're just saying for the most critical patients. And that's why I think it'll be important to figure out that rating system… because you're right, like somebody shouldn't be waiting 15 years, for sure not. But if somebody is in critical, you know, almost about to die, how can we justify, like I know, Jane is sitting for 10 years, you know, on dialysis, but Jane is still able to have a transplant eventually.

In this excerpt, participants were discussing a scoring system, which would include consideration of multiple clinical factors, such as epitope compatibility, health status and time on waitlist to determine priority for kidney transplantation – that could provide the flexibility needed to balance various concerns and values simultaneously. Other suggestions included identifying a “tipping point” at which a candidate should be prioritized, or specifying a maximum percentage of kidneys that should be reserved for exceptional cases.

This need for Flexibility was particularly important to participants in the context of epitope compatibility, where there are no large-scale empirical studies of clinical utility/improved outcomes compared to the current kidney transplantation allocation criteria. Without concrete outcomes data, the pursuit of Health Maximization through the introduction of epitope compatibility is uncertain, and participants expressed caution around its implementation. They described wanting epitope compatibility to be introduced in a “balanced” manner to uphold the values of Fairness and Protection/Mitigation of Negative Impacts, especially until sufficient evidence of utility is acquired and Health Maximization can be pursued more confidently (i.e., basing health policy decisions on the current science/evidence base):

Evelyn (session 3): To me I couldn’t like sleep at night thinking somebody could possibly die because of the new system and it may not, in the long run, really lead to a longer kidney health for people. So, this way it’s additional criteria [considered in kidney allocation], we can monitor people over the life span of their kidney and make sure that this really does work… Put it as a criteria because we have some science background but then we also have the [existing] system in place so we're not just putting epitope [compatibility] as the top priority or criteria, so to me it's a balanced way to do it.

The need for Flexibility drove the formation of six of the nine recommendations (see Fig. 1), demonstrating the high importance that participants placed on upholding Fairness and Protection/Mitigation of Negative Impacts for individual patients (or groups of patients), while also using science to pursue Health Maximization for the good of society.

The remaining recommendations (7-9) were driven by the needs for a Transition Plan, Transparent Communication and Accountability. Each of these principles were ways to uphold several values (Fig. 1, Table 4). All three, however, included the responsibility to maintain public trust in the kidney allocation system, in order for the public “to buy in and accept this kind of change [the introduction of epitope compatibility]”. If trust was lost, participants foresaw “social backlash”, “bad press” and a potentially detrimental impact on kidney donation and transplantation rates. Participants discussed the importance of trust multiple times throughout the deliberation, and these discussions were less contentious and time-consuming than discussions around Fairness and the need for Flexibility; rather, participants focused on articulating the specific ways in which trust could be maintained or supported (Recommendations 7–9). Support for these recommendations was nearly unanimous, suggesting that the need to maintain trust was more easily supported by participants, and that trust was required in addition to (rather than conflicting with) participants’ other values.

Two values – Efficient Use of Resources and Logic/Rationality – did not enter into the discussion of operational principles, but did arise in the assessment of recommendations. For example, several participants voted against Recommendations 3–5 because of their value for Efficient Use of Limited Resources: the belief that prioritizing other factors over epitope compatibility would result in wasted kidneys and healthcare resources, due to poor survival rates and/or re-transplantations. In addition, Recommendation 4 (prioritizing quality of life) was unsupported by the majority of participants who struggled to endorse a concept that could not be objectively defined and measured, and that may encompass numerous factors besides one’s medical condition (e.g., geographic location/distance from dialysis clinic, impact on one’s work and activity levels, family life). This demonstrated participants’ value for Logic/Rationality – focusing on factors that were objective and measurable—in some aspects of kidney allocation decisions.

## Discussion

This is the first study to report on public preferences and values related to epitope compatibility for deceased donor kidney allocation. The nine recommendations formed in this online Canadian public deliberation were driven by five values: Health Maximization, Protection/Mitigation of Negative Impacts, Fairness, Science/Evidence-based Healthcare, and Responsibility to Maintain Trust in public healthcare systems. Conflicts between these values resulted in operational principles—Flexibility, Accountability, Transparent Communication and the need for a Transition Plan – that would be required for the introduction of epitope compatibility in a publicly acceptable manner. Each of these principles enabled the maintenance or balancing of participants’ values against one another, and directly drove the formation of all nine recommendations.

Much of participants’ discussion centered around the conflict between their value for Health Maximization – potentially enabled by the use of epitope compatibility – and the values of Fairness and Protection/Mitigation of potential negative impacts of such initiatives. This conflict is unsurprising given the numerous reports in the solid organ transplant literature of public desire to maximize successful outcomes [11, 12, 14–17], while also prioritizing medically urgent cases [11–13, 15], in order to prevent fatal consequences or further organ damage; in addition to maintaining fair or equitable access to transplantation [11, 12]. In this public deliberation, participants perceived greater potential for both improved transplant outcomes, and for some candidates to be disadvantaged – with rapidly declining health and/or quality of life – while waiting for an epitope compatible kidney. Despite previous research demonstrating that wait time is a priority [12, 16, 17] and the tendency to prefer waitlist-based programs [10], participants did not construct a recommendation around wait times – though many participants were uncomfortable with long waiting times and felt this outcome would also be unfair. The notion of Fairness underpinned the majority of participants’ deliberation and contributed to eight of the nine recommendations (via the operational principles), and there was significant complexity within participants’ interpretation of Fairness. It seems that certain types of negative or unfair impacts (declining health) are perceived as critical and must, therefore, be mitigated against while pursuing maximum benefit from donor kidneys through the use of epitope compatibility.

The tension and potential trade-offs between these values – Health Maximization, Fairness and Protection/Mitigation of Negative Impacts—culminated in the need for Flexibility in kidney allocation policy, which drove the majority of participant recommendations (1–6 of 9). Flexibility was particularly important in the context of epitope compatibility, in which not all epitope mismatches cause an antibody response [26], and the evidence for clinical utility is promising, but based on retrospective studies [7]; thus, a particularly nimble approach is necessary to adjust for emerging biological and clinical evidence. The need for Flexibility is also consistent with a review of public preferences which concluded that no single criterion should be used as an overriding principle for organ allocation [15]. Flexibility has already been utilized in multiple organ allocation systems in the form of weighted points-scoring systems [10, 27], suggesting that this may be a publicly acceptable and balanced way in which epitope compatibility could be introduced.

Participants were clear that Transparent Communication and Accountability should be maintained throughout the implementation of epitope compatibility into kidney transplantation programs. The principles of transparency and accountability are crucial in kidney allocation policies [10, 28], particularly in that they maintain trust between healthcare systems and the public – the ultimate end user and also supplier of donor kidneys. Thus, while accountability and transparency have already been prioritized in current allocation systems [28], the maintenance of public trust will be critical whenever changes to the criteria are being considered. This may be especially important with epitope compatibility – a novel, cutting-edge technique that would impact the kidney offer order and waiting time. Again, the fact that clinical evidence for epitope compatibility is evolving [6, 29–32] – which raised concern for members of the deliberation who value Evidence-based Healthcare—heightens the need for Accountability to ensure that new policies perform as projected and result in benefit for transplant recipients and society more broadly.

Two values – Efficient Use of Resources and Logic/Rationality—did not contribute to the operational principles, but were raised as concerns by some participants. This was particularly relevant in discussions regarding quality of life, which has been discussed in terms of its effect on medical urgency (pre-transplant quality of life) as well as potential benefit from transplantation (expected post-transplant quality of life) [15]. However, there is little information regarding public preferences for quality of life in transplant allocation decisions, largely due to the subjective interpretation of what quality of life entails [15]. Given the complex trade-offs made necessary by the allocation of a limited resource, our participants focused their recommendations on measurable outcomes (such as health status, as determined by clinical expertise), which will allow for effective outcomes monitoring of new kidney allocation policies.

Participants were keen to pursue epitope compatibility – cautiously, with built-in safeguards – despite the absence of conclusive, prospective clinical outcomes data. While clinical trials may be challenging to pursue, several participants expressed that the implementation of epitope compatibility could actually help provide the required evidence for epitope-compatible transplantation outcomes. This may suggest, along with Recommendation 9 (which called for regular assessment and communication of epitope compatibility outcomes), that the implementation of policy with planned evaluation of the policy and its implications, along with appropriate informed consent [33], may be worth examining further as a possible future direction.

Public trust – which can be supported by transparency and communication [34]—was a key value of participants in this deliberation. The need to do right by the public is particularly emphasized in the context of limited resources (organs available for transplantation) that may confer a risk of inequities, and in universal healthcare systems [35] such as Canada’s, which is expected to fulfill a fiduciary duty to protect its vulnerable citizens [36]. The implementation of epitope compatibility for deceased donor kidney allocation will require the understanding and incorporation of public values and expectations in order to prevent a loss of credibility [5] and trust [34] that could hinder the potential for improved population health outcomes.

### Limitations

The results of this public deliberation are context-specific, and may not be generalizable to other settings: participants were recruited from the Canadian population and provided with background information (via written material and expert speakers) to inform their discussions. Our analysis focused specifically on the values manifested in participants’ final recommendations; thus, the present results may not may not encompass all values that may be considered by the public with regards to the use of epitope compatibility for deceased donor kidney allocation.

The online format of the deliberation was significantly shorter than the typical four-day in-person event, which may have impacted the depth of participant discussions. However, given the format of public deliberation—which specifically asks participants to consider alternative perspectives in order to collaboratively construct recommendations—the values reported here are informed by a breadth of different perspectives and types of reasoning by diverse representatives of the Canadian public.

Despite efforts to include a greater diversity of participants, the majority of our cohort was White and we recognize that self-selection bias is a feature of our sample and a limitation. Efforts to address this shortcoming were complicated by the timing of the deliberation related to the Covid-19 pandemic and the need to conduct the deliberation online. Future research with in-person deliberations would provide more opportunity to explore the equity and diversity dimensions of epitope compatibility in deceased donor kidney allocation.

## Conclusions

This online public deliberation provides a set of recommendations to be considered in policy decisions about epitope compatibility in deceased donor kidney allocation. Qualitative analysis identified the values – Health Maximization, Protection/Mitigation of Negative Impacts, Fairness, Science/Evidence-based Healthcare, and the Responsibility to Maintain Trust – that underly public recommendations for the introduction of epitope compatibility. These values can be upheld by incorporating Flexibility, Accountability, Transparent Communication, and a Transition Plan into future deceased donor kidney allocation policy changes.

## Supplementary Information

**Additional file 1.**

**Additional file 2.**

## Data Availability

The data used during the current study are not publicly available in order to protect the confidentiality of participants. Deidentified data may be available from the corresponding author on reasonable request.

## Abbreviations

HLA: Human Leukocyte Antigen
PM: Precision Medicine
