# Supplementary material for: Public values and guiding principles for implementing epitope compatibility in kidney transplantation allocation criteria: results from a Canadian online public deliberation
Source: BMC Public Health. 2023 May 10;23:844. doi: 10.1186/s12889-023-15790-w (PMC10170053; doi:10.1186/s12889-023-15790-w)
Supplement: Supplementary file 1 — Additional file 1. [file 12889_2023_15790_MOESM1_ESM.docx]

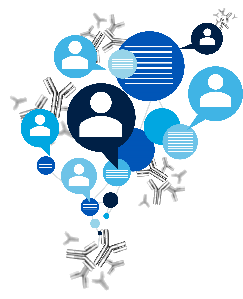


Kidney Transplant Deliberation

**Kidney Transplant Deliberation**

**Considering new criteria for kidney transplantation: A public deliberation**

Agenda for November 6, 9-16, 20, 27 and December 4, 2021

NOTE: All times listed are in Pacific time

**SESSION 1: November 6th**

9:00 AM Pre-meeting debrief with team

9:55 AM Log-in to Zoom session

10:00-10:10 AM Welcome address, territorial acknowledgment

10:10-10:30 AM Participant and research team introductions, acknowledge observers

10:30-10:50 AM Overview of the event and ground rules

10:50-11:55 AM Expert speaker presentations, Q&A

1) Kidney transplant doctor in BC

2) Bioethicist

3) Indigenous knowledge keeper and elder

4) Patient perspective

11:55-12:00 PM Overview of tasks and goals for Session 2

**SESSION 2: November 9 (3-5pm), 10 (6-8pm), 13-14 (10am-12pm), 15 (5-7pm), 16 (4-6pm)**

9:55 AM Log-in to Zoom session

10:00-10:30 AM Welcome & more in-depth introductions (“What’s your favourite pastime?”)

10:30-10:35 AM Introduction to Hopes and Concerns task

10:35-11:35 AM Group discussion: Hopes and Concerns

11:35-11:55 AM Deliberation practice question & voting (e.g., “Canada should retain the current system daylight savings time.”)

11:55-12:00 PM Overview of tasks and goals for Session 3

**SESSION 3: November 20th**

9:55 AM Log-in to Zoom session

10:00-10:05 AM Welcome, session overview, acknowledge observers

10:05-10:25 AM Review Hopes and Concerns from Session 2

10:25-11:50 AM Deliberation on Question 1, recommendations, voting

11:50-12:00 PM Overview of tasks and goals for Session 4

**SESSION 4: November 27th**

9:55 AM Log-in to Zoom session

10:00-10:05 AM Welcome, session overview, acknowledge observers

10:05-10:25 AM Recap of Question 1

10:25-11:50 AM Deliberation on Question 2, recommendations, voting

11:50-12:00 PM Overview of tasks and goals for Session 5

**SESSION 5: December 4th**

9:55 AM Log-in to Zoom session

10:00-10:05 AM Welcome, session overview, acknowledge observers

10:05-10:20 AM Policy panel introductions

10:20-10:50 AM Review full list of recommendations

10:50-11:50 PM Policy panel discussion and questions

11:50-12:00 PM Wrap up and next steps – will analyze and write this up, will share recommendations with participants (shared in a follow-up report)
