# Supplementary material for: Public values and guiding principles for implementing epitope compatibility in kidney transplantation allocation criteria: results from a Canadian online public deliberation
Source: BMC Public Health. 2023 May 10;23:844. doi: 10.1186/s12889-023-15790-w (PMC10170053; doi:10.1186/s12889-023-15790-w)
Supplement: Supplementary file 2 — Additional file 2. [file 12889_2023_15790_MOESM2_ESM.docx]

Supplementary material: Codebook

| **Code** | **Definition/Explanation** |
| --- | --- |
| **Deductive codes** (reference 23) | |
| Beneficence | Obligation to produce benefit, for individual patients; involves active contribution towards the welfare of others |
| Equity/justice | Demands equal opportunities. This includes a fair distribution of health outcomes in societies, often discussed as “health equity” |
| Non-maleficence | Do no harm; involves the omission of harmful action; expressing concern for harm resulting (but no expression of action to be taken) |
| Health maximization | Focus on maximizing population health; most benefit for the most people |
| Efficiency | Moral duty to use scarce health resources efficiently |
| Respect for autonomy | Every person has a high value – qua their autonomy – and cannot merely be treated as a means to the end of others’ good |
| Proportionality | Demands that in weighing and balancing individual freedom against wider social goods, considerations will be made in a proportionate way; individual welfare vs. collective benefit in health |
| **Inductive Codes** | |
| Fairness | Standardized treatment such that no person is unduly favoured or disadvantaged |
| Protection/mitigation | Suggestion to act or put in place strategies to protect the vulnerable |
| Responsibility | To use resources well, or to maintain trust in the public healthcare system |
| Accountability | Ongoing monitoring/assessment of healthcare policies/decisions; being accountable to the public |
| Science or evidence-based healthcare | Obligation of using health interventions shown to result in benefit for patients |
| Clinical expertise | Value for physicians’ clinical judgement/decision-making |
| Individuality | Right to personal treatment, case-by-case evaluation |
| Ensuring quality of life | Consideration of and priority attributed for ensuring quality of life for patients |
| Flexibility | In health system, in decision-making |
| Clarity/certainty of information | Importance of having certainty (or reducing/minimizing uncertainty) in health-related information and decision-making |
| Trust | From patient’s perspective; trust in healthcare decision-making and clinicians |
| Logic/rationality | Using a scientific, logical, mathematical or otherwise rational approach to problem-solving or decision-making |
